# High temperatures are associated with decreased immune system performance in a wild primate

**DOI:** 10.1126/sciadv.adq6629

**Published:** 2024-11-29

**Authors:** Jordan M. Lucore, Jacinta C. Beehner, Amy F. White, Lorena F. Sinclair, Vasco Alexandre Martins, Sarah A. Kovalaskas, Juan Carlos Ordoñez, Thore J. Bergman, Marcela E. Benítez, Andrew J. Marshall

**Affiliations:** ^1^ Department of Anthropology, University of Michigan, Ann Arbor, MI, USA.; ^2^ Capuchinos de Taboga Research Project, Taboga Forest Reserve, Guanacaste, Costa Rica.; ^3^ Department of Psychology, University of Michigan, Ann Arbor, MI, USA.; ^4^ Department of Anthropology, Durham University, Durham, UK.; ^5^ Department of Anthropology, Emory University, Atlanta, GA, USA.; ^6^ Department of Ecology and Evolutionary Biology, University of Michigan, Ann Arbor, MI, USA.; ^7^ Program in the Environment, University of Michigan, Ann Arbor, MI, USA.; ^8^ School for Environment and Sustainability, University of Michigan, Ann Arbor, MI, USA.; ^9^ Program in Computing for the Arts and Sciences, University of Michigan, Ann Arbor, MI, USA.

## Abstract

Rising temperatures due to climate change are predicted to threaten the persistence of wild animals, but there is little evidence that climate change has pushed species beyond their thermal tolerance. The immune system is an ideal avenue to assess the effects of climate change because immune performance is sensitive to changes in temperature and immune competency can affect reproductive success. We investigate the effect of rising temperatures on a biomarker of nonspecific immune performance in a wild population of capuchin monkeys and provide compelling evidence that immune performance is associated with ambient temperature. Critically, we found that immune performance in young individuals is more sensitive to high temperatures compared to other age groups. Coupled with evidence of rising temperatures in the region, our results offer insight into how climate change will affect the immune system of wild mammals.

## Teaser

Decreased immune system performance during periods of hot weather indicates physiological risk of climate change for wild animals.

## INTRODUCTION

Human-induced climate change is affecting the health of our planet in many devastating ways. Increasing temperatures are predicted to have major effects on wild animal populations and are frequently cited as an important threat to their persistence (*1*, *2*). There is evidence across a diversity of taxa that climate change is already affecting species distributions (*3*, *4*), behavior (*5*), and physiology (*6*, *7*), yet there has been little evidence that temperature changes have direct effects on individual fitness. The immune system, one of the body’s main defense mechanisms against infection, is an ideal avenue in which to assess the effects of rising temperatures because it is sensitive to changes in ambient temperature (*8*) and is an important determinant of individual fitness outcomes. Many hypotheses posit the negative effects of climate change on immune function (*9*, *10*); however, a relationship between the immune system and temperature has yet to be demonstrated in any wild mammal. Here, we provide compelling evidence that immune performance is associated with temperature in a natural population of tropical mammals and discuss the implications for population health in the face of climate change. Specifically, we examine how a marker of immune performance is affected by extended periods of high temperatures in a population of wild capuchin monkeys in Costa Rica.

The consequences of rising temperatures have primarily been observed in ectotherms because they lack thermoregulatory mechanisms to buffer the negative effects of thermal load on physiological performance (*11*, *12*) and, by extension, growth and reproduction (*7*). Thermal performance curves are often used to illustrate how physiological performance changes across a temperature range. Performance steadily increases with temperature until reaching a thermal optimum, beyond which performance quickly decreases (*13*). System performance can become heat-compromised at the far end of the temperature range due to anaerobic conditions created at high temperatures (*7*, *14*). Less is known about the relationship between physiological performance and temperature in endotherms because the effects of ambient temperature are buffered by thermoregulatory mechanisms. However, we know that temperature is an important predictor of immune performance because decreases in immune performance at high temperatures are well documented in livestock under experimental conditions [e.g., cows (*15*) and chickens (*16*)]. No comparative work exists on wild endotherms, particularly tropical species that are predicted to be vulnerable to shifts in temperature because they are adapted to a narrower temperature range than species at higher latitudes (*17*, *18*).

The immune system consists of two main branches: nonspecific immunity (i.e., innate resistance to infection) and adaptive immunity (i.e., acquired resistance to specific pathogens). Here, we focus on nonspecific immunity because it is the first line of defense against infection and deploys an immediate, nonspecific defense while adaptive immunity builds a pathogen-specific response (*19*, *20*). Nonspecific immunity is the primary line of defense for infants and juveniles while the rest of their immune system develops (*21*). Therefore, nonspecific immune competency in early life has implications for long-term health outcomes (*22*). Neopterin is an excellent way to measure the nonspecific immune system performance because it reflects the activation of nonspecific immune cells (*23*) and can be collected noninvasively from urine (*24*, *25*). Specifically, neopterin is excreted by macrophages and monocytes stimulated by interferon-γ produced by the T helper 1 (T_H_1) cellular immune response during the initial stages of infection (*23*, *26*). From here on, we define immune performance as T_H_1 cell activation of macrophages and monocytes.

Urinary neopterin has been used to monitor disease status (*27*) and immune ontogeny (*25*, *28*). However, no current studies have used neopterin to measure the effects of abiotic conditions on immune performance. Here, we examine changes in urinary neopterin (*n* = 670 samples) in 54 wild white-faced capuchin monkeys (*Cebus imitator*) to evaluate the relationship between temperature and immune performance. Air maximum and minimum temperatures were collected every 15 min and averaged 15 days before sample collection to evaluate how long-term temperature trends are associated with neopterin concentration. We also evaluated age-specific trends of temperature and neopterin using 2 years of longitudinal sampling of individuals ranging from 1 month to 36 years of age. To situate our results within the larger context of climate change, we show evidence of rising temperatures in Northwestern Costa Rica using a global atmospheric model (*29*).

## RESULTS

**Fig. 1.**
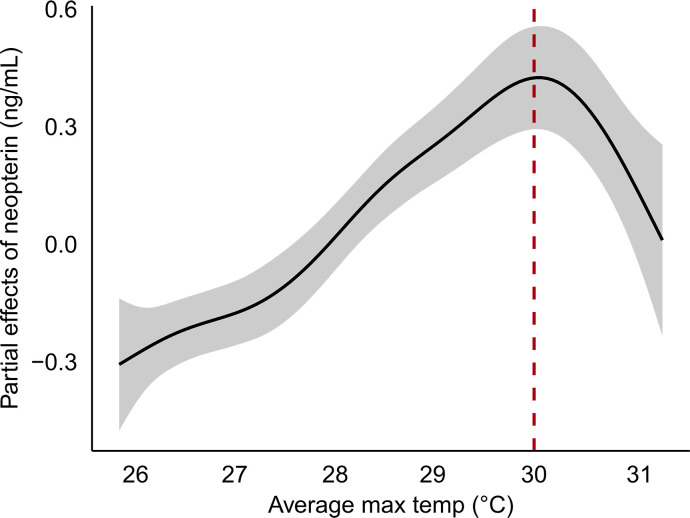
Relationship between neopterin and temperature. Partial effects plot of the generalized additive model that evaluates the nonlinear relationship between neopterin concentration and average maximum temperature in the 15 days before sample collection. The relationship resembles a thermal performance curve where the dashed red line indicates the thermal optimum for immune performance after which we see a steep decline in performance.

We found a relationship between maximum temperature and neopterin in capuchin monkeys that resembles a thermal performance curve (*11*–*13*) where neopterin concentration increases with temperature until reaching a thermal optimum, at 30°C, beyond which neopterin concentration quickly decreases (Fig. 1). Modeling the relationship with minimum temperature as a predictor yielded the same results (fig. S1); therefore, we will report the results for maximum air temperature only. The steep decline in neopterin between 30° and 31°C is typical of a thermal performance curve where performance drops quickly after passing the thermal optimum. The practically linear relationship between neopterin and temperature between 24° and 30°C corroborates in vitro studies where rising temperatures increase immune performance (*30*, *31*).

**Fig. 2.**
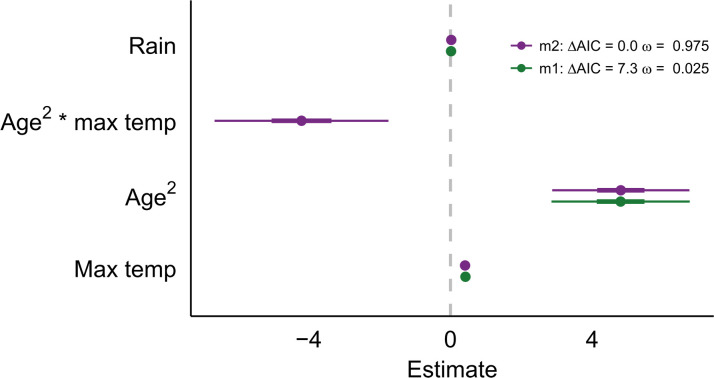
Model results for neopterin. Comparison of generalized linear mixed models for models with greater than >0.001% of the model weight. Similar results are seen across models. Thick bars represent 50% CI, and thin bars represent 95% CI. Both models support the U-shaped relationship between neopterin and age. Model 2 holds the most model weight and includes the reliable interaction effect between age^2^ and maximum temperature. Rain shows no reliable effect.

**Fig. 3.**
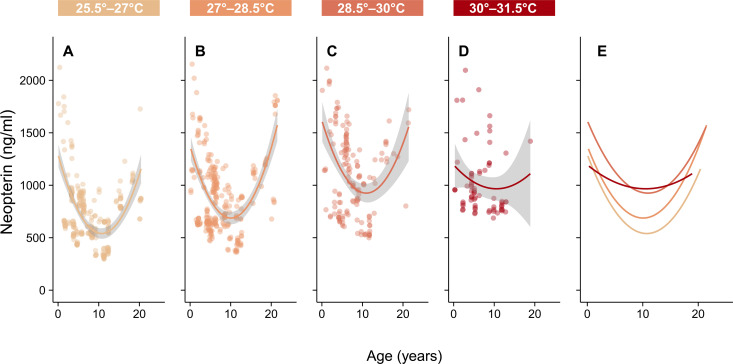
Visualization of the interaction effect between age and temperature. The relationship between age and neopterin concentration is plotted across four, discrete temperature ranges. (**A** to **D**) The top panel shows the discrete temperature bins where each bar lists the temperature cutoffs for its corresponding plot. The bars become increasingly dark to indicate rising temperatures. The predictions from the best-fitting generalized linear model are plotted in the color of the corresponding bar that lists the temperature range for each plot. Gray shading represents 95% confidence intervals. (**E**) shows the same curves from (A) to (D) overlapping to emphasize the flattening of the U-shaped neopterin-age relationship at temperatures above 30°C, particularly in young individuals.

Neopterin shows a U-shaped relationship with age (β_age2_ = 4.81, Fig. 2) where increased levels in early life indicate a reliance on nonspecific immunity (*21*), and increased levels in late life are characteristic of immune system senescence (*32*). We found that the relationship between temperature and neopterin is also associated with age; in our best-fitting model, temperature shows a reliable negative interaction effect with age (β_temp*age2_ = −4.20, Fig. 2). To interpret the interaction effect between a polynomial and continuous predictor, we plotted the U-shaped relationship between age and neopterin across discrete temperature bins (Fig. 3). Similar to the thermal performance curve, neopterin concentration increases across all ages up to 30°C (Fig. 3, A to C). Above 30°C, there is a flattening of the curve, particularly on the left side depicting young individuals (Fig. 3D), which is emphasized when the curves are superimposed on each other (Fig. 3E). We found no reliable effect of sex, reproductive state, or season (as measured by average rainfall; β_rain_ = 0.02) on neopterin (fig. S2 and table S5), indicating that the relationship between neopterin and temperature is not dependent on seasonal temperature changes.

**Fig. 4.**
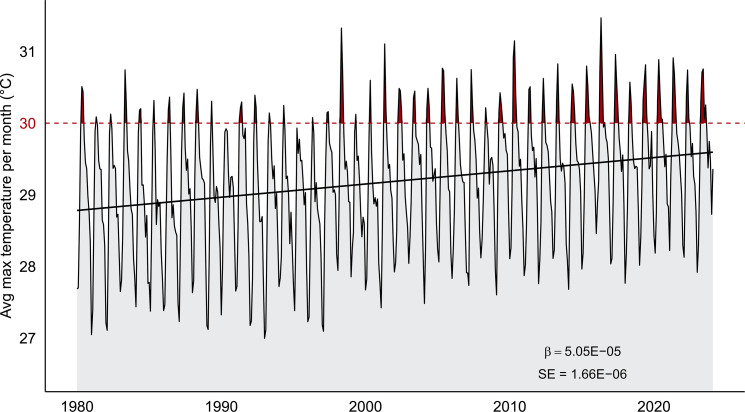
Climate change in Northwestern Costa Rica. Longitudinal change in average monthly maximum temperature over the last 43 years visualized using data from the MERRA-2 global atmospheric dataset. The black line represents a linear model that shows an increase in daily maximum temperature over time. The horizontal red line at 30°C represents the thermal optimum for immune performance after which we see a steep decline in performance. The red shading highlights the increasing number of days and longer periods above 30°C.

Temperatures are rising in Northwestern Costa Rica (Fig. 4), showing an average increase of 0.18°C each decade since 1980 (β_date_ = 5.05e−05, SE = 1.66e−06). Rising temperatures have resulted in more days and longer periods where temperatures are above 30°C, the thermal optimum for T_H_1 cellular immune performance in our study population. For example, between 1980 and 2014, only 18% of 20 days recorded average temperatures above 30°C, compared to the last decade when the number increased to 34%. This equates to an 89% increase in the number of 30°C-plus days in the last decade.

## DISCUSSION

Here, we provide compelling evidence that immune performance is associated with temperature in a natural mammalian population and show that immune performance in young individuals is more sensitive to high temperatures than other age groups. Average temperatures above 30°C for an extended period, at least 15 days, are associated with a steep decline in neopterin concentration (Fig. 1). The decline in neopterin concentration after 30°C is most pronounced in young individuals, indicating that immune performance in early life is especially affected by high temperatures (Fig. 3). Given that temperature was modeled as a 15-day average, these results represent the association between neopterin and extended periods of elevated temperature during which behavioral (*33*) and physiological (*34*) adaptations to cope with short-term heat stress are likely less effective at buffering thermal load. Coupled with evidence of rising temperatures in the region (Fig. 4), our results offer some insight into how climate change could affect the immune systems of wild mammals.

Thermal performance curves are rarely observed outside of laboratory conditions where ecological variables can influence the temperature-performance relationship (*35*). Despite this, the relationship between neopterin and temperature resembles a thermal performance curve, indicating the importance of temperature in determining immune performance in wild populations. The thermal optimum at 30°C is not necessarily physiologically optimal because it appears to be driven by temperature as opposed to infection or other challenges that require an immune response. Further, because system performance can become heat-compromised at the far end of the temperature range, the observed drop in performance after 30°C could indicate that this population may soon, or already, experience periods where T_H_1 cellular immune activation is heat-compromised (*12*). White blood activation is a critical defense mechanism in response to initial infection; impaired activation could reduce immune competency, increase instances of infection, and affect long-term health outcomes. This trend is specifically concerning in our study population because white-faced capuchins in Northwestern Costa Rica are endemic to tropical dry forests that are highly seasonal (*36*); therefore, they are likely adapted to a wider temperature range compared to many tropical specialists (*33*, *34*). Given the evidence that our study population could be nearing the limit of their thermal range, it is likely other tropical species are experiencing more severe effects of heat-compromised performance.

High neopterin in young individuals indicates reliance on nonspecific immunity in early life while the adaptive immune system develops (*21*). The decline in neopterin over the first 5 years of life is typical of other nonhuman primate species (*28*) (Fig. 3, A to C) and likely represents the period of development for the adaptive immune system. Here, we show that the neopterin-temperature relationship is associated with age (Fig. 2) where young individuals have lower neopterin than expected at high temperatures compared to other age groups (Fig. 3). Young individuals may reach their thermal optimum at lower temperatures or may exhibit a steeper decrease in performance after 30°C compared to other age groups. Under both scenarios, young individuals likely approach heat-compromised T_H_1 cell activation faster than older individuals, indicating that immune performance in early life is especially affected by high temperatures. Heat-compromised white blood cell activation in early life could leave individuals vulnerable to infection because the other branches of their immune system remain undeveloped. Lack of immune competency in early life is associated with higher risks of infection (*22*) and stunting (*37*), which can negatively affect reproductive success (*38*) or result in death before reproductive age (*39*). Therefore, the susceptibility of young individuals to heat-compromised immune performance may be an important mechanism by which rising temperatures due to climate change could increase extinction risk through direct effects on individual fitness.

The temperature-neopterin relationship does not appear to be a product of seasonal shifts in temperature or confounding factors associated with seasonality. Seasonal changes in tropical dry forests are associated with shifts in other factors (e.g., parasite load and resource scarcity) that can affect immune performance either directly or indirectly by stressing other physiological processes. However, neopterin is not associated with rainfall (Fig. 2), which is a reliable measurement of season in tropical dry forests (*40*). We acknowledge that the lack of intracellular parasite measurements is a limitation of this study as parasites can show seasonal patterns of infection (*41*, *42*) and have been associated with neopterin concentrations (*28*). However, the relationship between parasitic infection and neopterin is not straightforward (*28*, *43*) and requires further research. Given that climate change is affecting the emergence and distribution of infectious diseases (*44*, *45*), understanding the link between temperature, parasites, and immune performance will likely help predict long-term health outcomes in the coming decades.

Temperatures are rising and will result in longer periods where temperatures are above the thermal optimum for immune performance in our study population. In the last decade, the number of days above 30°C in Northwestern Costa Rica showed an 89% increase compared to the previous three decades (Fig. 4). This trend is expected to continue, resulting in an overall temperature increase between 4° and 6°C by 2080 (*46*). Given the steep decline in immune performance between 30° and 31.5°C, a 4° to 6°C increase promises periods where immune performance is heat-compromised. It is important to acknowledge that the immune system deploys multifaceted and redundant mechanisms (*20*) of which we have only measured one, and it is, therefore, difficult to predict the effects of heat-compromised T_H_1 immune activation on long-term health outcomes with much certainty. However, given that diverse immune mechanisms have been shown to respond to temperature (*8*, *15*, *30*), it is likely that rising temperatures due to climate change could affect other branches of immunity and likely compound negative health outcomes. Future research would benefit from the collection of additional immune biomarkers from naturally occurring populations to gain a broader understanding of how temperature affects overall immune performance in wild mammals. Long-term research is also necessary to identify and quantify the effects of heat-compromised immune performance on individual fitness outcomes, as this could be a mechanism by which climate could increase extinction risk in diverse taxa. As temperatures continue to increase at faster rates, we expect species will continue to experience more diverse and severe physiological effects due to our changing—and increasingly challenging—climate.

## MATERIALS AND METHODS

### Site information and sample collection

Between September 2021 and August 2023, we collected 676 urine samples from three habituated groups of wild *C. imitator* associated with the Capuchinos de Taboga research project in the Taboga Forest Reserve. The Taboga Forest Reserve is a 516-ha tropical dry forest in the Guanacaste region of Northwestern Costa Rica that is owned by the Universidad Técnica Nacional. It is an important piece of a fragmented biological corridor connecting the Tempisque River Basin to the Guanacaste Mountains (*47*). We collected samples from 54 individually identified individuals ranging from 1 month to 35 years of age. For individuals younger than 7 years, age was calculated by birth date; for individuals older than 7 years, age was estimated based on body condition by one of us (J.C.O.) with more than two decades of experience observing wild white-faced capuchins. We collected samples opportunistically, from individuals that appeared healthy upon visual inspection, using a clean catch method (*48*) to avoid rainfall and fecal contamination between the hours of 5:00 a.m. and 6:00 p.m. We collected samples from leaves or the ground only when they did not appear contaminated with soil or feces. We then transferred the sample to 1.8-ml tubes using disposable pipets and immediately stored samples in a portable cooler on ice for up to 7 hours. We stored the samples in a −20°C freezer at the end of the field day.

### Sample analysis

We measured the neopterin concentration of all samples using a commercially available competitive exclusion neopterin enzyme-linked immunosorbent assay (Neopterin ELISA, Ref. RE59321, IBL International GMBH, Hamburg, Germany) in the Capuchinos de Taboga field laboratory. The kit was developed to detect neopterin concentration in human serum, plasma, and urine, but has been validated for use in the wild *C. imitator* population associated with the Capuchinos de Taboga research project (*25*). We thawed, centrifuged, and aliquoted all samples to remove precipitate and measured sample water volume using a handheld refractometer (Aichose, Ref. SR0021-ATC) to control for sample concentration. We then diluted samples to 1:64 with manufacturer assay buffer and ran the assay using the manufacturer protocol. We reran samples with a coefficient of variation higher than 15.5%. We rediluted and reran samples with low binding (>85% binding) or high binding (<15% binding) on the standard curve. The inter-assay variation for 26 plates was 9.48%.

### Temperature observations

To evaluate the relationship between neopterin concentration and temperature, we used observations from the Palo Verde meteorological station located at the Organization for Tropical Studies (OTS) Palo Verde research station, 10.345°N, 85.339°E, approximately 20.81 km southwest of the Taboga Forest Reserve. Palo Verde is one of the last remaining fragments of tropical dry forest in Central America similar to the Taboga Forest Reserve (*49*). We used the OTS Palo Verde climate data due to habitat similarity and close geographical location to our study site. The OTS Palo Verde meteorological station records maximum and minimum air temperature every 15 min.

Because the OTS Palo Verde climate data are only available from 2007 onward, we used the Modern-Era Retrospective analysis for Research and Applications, Version 2 [MERRA-2 (*29*)] developed by the NASA Goddard Space Flight Center and Global Modeling and Assimilation Office to evaluate long-term temperature trends in the Guanacaste region. MERRA-2 is a global atmospheric dataset that provides numerous meteorological variables on a horizontal grid with a spatial resolution of 0.625° × 0.5° longitude-latitude. We extracted the average daily maximum air temperature at 2 m above surface level from the years 1980 through 2023 at 10.5°N, 85.625°E, the closest available point on land to the OTS Palo Verde meteorological station. To ensure the two datasets were comparable, we standardized the MERRA temperatures by calculating the deviation from the mean temperature of the Palo Verde data and adding the deviation to the MERRA data.

### Statistical analysis

For our neopterin data, we removed three samples that were 4 SD above the mean before conducting our analysis. We also removed three samples associated with the oldest individual in our population (34 years of age at the beginning of the study) to standardize the age spread across social groups. Excluding this individual did not change our results (tables S1 to S3).

To determine the best-fitting temperature predictor for neopterin concentration, we averaged maximum temperature (measured every 15 min) at different time intervals before sample collection. We then built a group of generalized additive models (GAMs) in which each model included a different temperature variable as a predictor. Neopterin concentration was the outcome variable for all models. We also included predictors for age and social group, which are known predictors of neopterin, and a random intercept for individual to control for repeated sampling. We scaled age and maximum temperature in all models for ease of coefficient interpretation. We fit age using a spline because of the known, nonlinear relationship between neopterin and age. We also fit maximum temperature using a spline because we are interested in finding the best-fitting, nonlinear relationship between maximum temperature and neopterin concentration. We fit social group as a linear variable because it was a discrete variable with three levels. We used a Gamma distribution with a log link function to fit the model because the outcome variable was continuous, positive, and left skewed with a long right tail. The best-fitting model included the predictor for the average maximum temperature of the 15 days before sample collection (table S4).

To compare maximum versus minimum temperature predictors, we reran this analysis using minimum temperature (measured every 15 min) and found the same result—the best-fitting model included the predictor for the average minimum temperature of the 15 days before sample collection. Model results were also congruent across all other time intervals (table S4). Further, the nonlinear relationship between maximum and minimum temperature and neopterin concentration was the same (fig. S1). Therefore, because there was no difference between temperature predictors, we chose to use the model including the average maximum temperature 15 days before sample collection to evaluate the nonlinear relationship between temperature and neopterin concentration using a GAM (*n* = 670 samples).

We evaluated the relationship between age, temperature, and immune function using multiple generalized linear mixed models and compared them using the Akaike Information Criterion corrected for small sample sizes (AICc) (*n* = 670 samples). Similarly, neopterin concentration was the outcome variable for all models, and we fit the models using a Gamma distribution with a log link function. All models included age, age^2^, social group, average rainfall, sex, and female reproductive status as predictors. Two models included maximum temperature as a predictor and only one model included an interaction effect between age^2^ and temperature (table S5 and fig. S2). To control for repeated sampling, we included a random intercept for individual. We scaled age, rain, and temperature in all models for ease of coefficient interpretation.

We used the MERRA-2 (*29*) data to assess long-term patterns in maximum temperature by fitting two linear mixed effect models with daily maximum temperature as the outcome variable (*n* = 16,103) and compared them using Akaike Information Criterion (AIC). We fit the models using a Gaussian distribution given the normal distribution of the outcome variable. We built the first model as an intercept model and compared it to the second model that included date as the predictor. We conducted all analyses in R version 4.3.1 (*50*).
