# Supplementary material for: High temperatures are associated with decreased immune system performance in a wild primate
Source: Sci Adv. 2024 Nov 29;10(48):eadq6629. doi: 10.1126/sciadv.adq6629 (PMC11619714; doi:10.1126/sciadv.adq6629)
Supplement: Supplementary file 1 — Figs. S1 and S2 Tables S1 to S5 [file sciadv.adq6629_sm.pdf]

Science Advances
AAAS

Supplementary Materials for

High temperatures are associated with decreased immune system performance in a wild primate

Jordan M. Lucore *et al.*

Corresponding author: Jordan M. Lucore, jlucore@umich.edu

Sci. Adv. **10**, eadq6629 (2024)
DOI: 10.1126/sciadv.adq6629

This PDF file includes:

Figs. S1 and S2
Tables S1 to S5

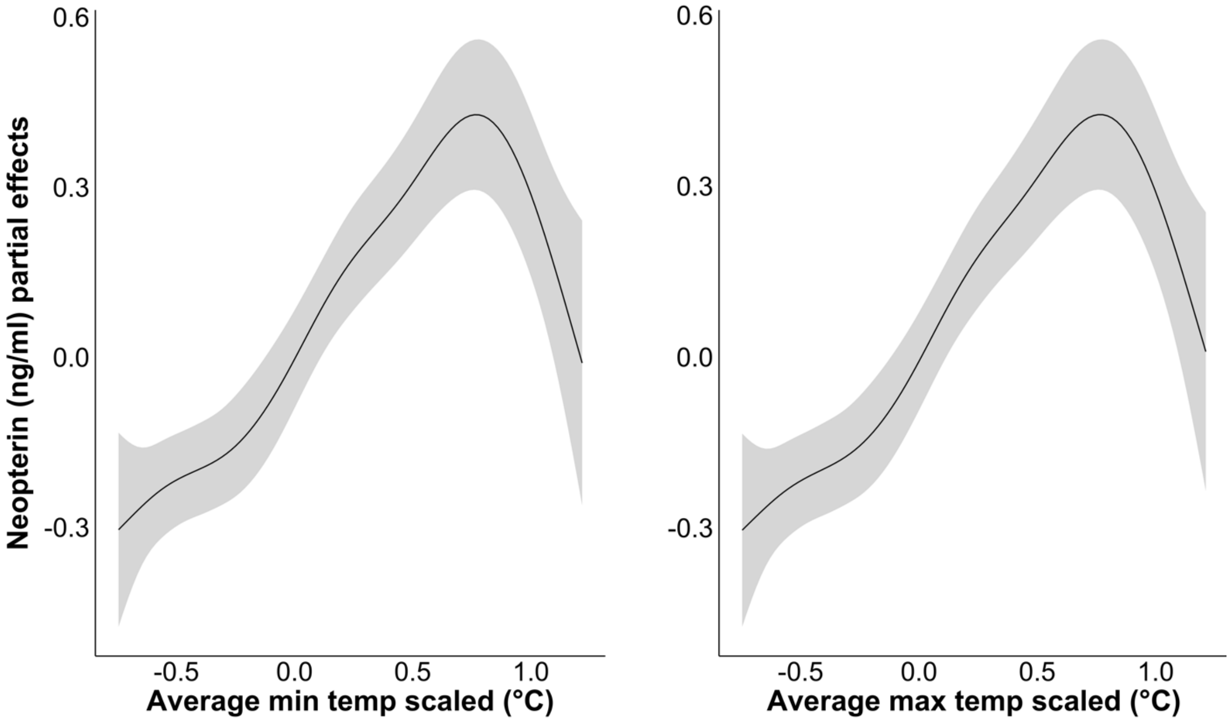

Fig. S1. Partial effects plot of the generalized additive models that evaluate the non-linear relationship between neopterin concentration and the average temperature in the 15 days before sample collection. The left plot visualizes the relationship between average minimum temperature compared to the right plot which illustrates the relationship between maximum temperature. Both temperature measurements yield the same results.

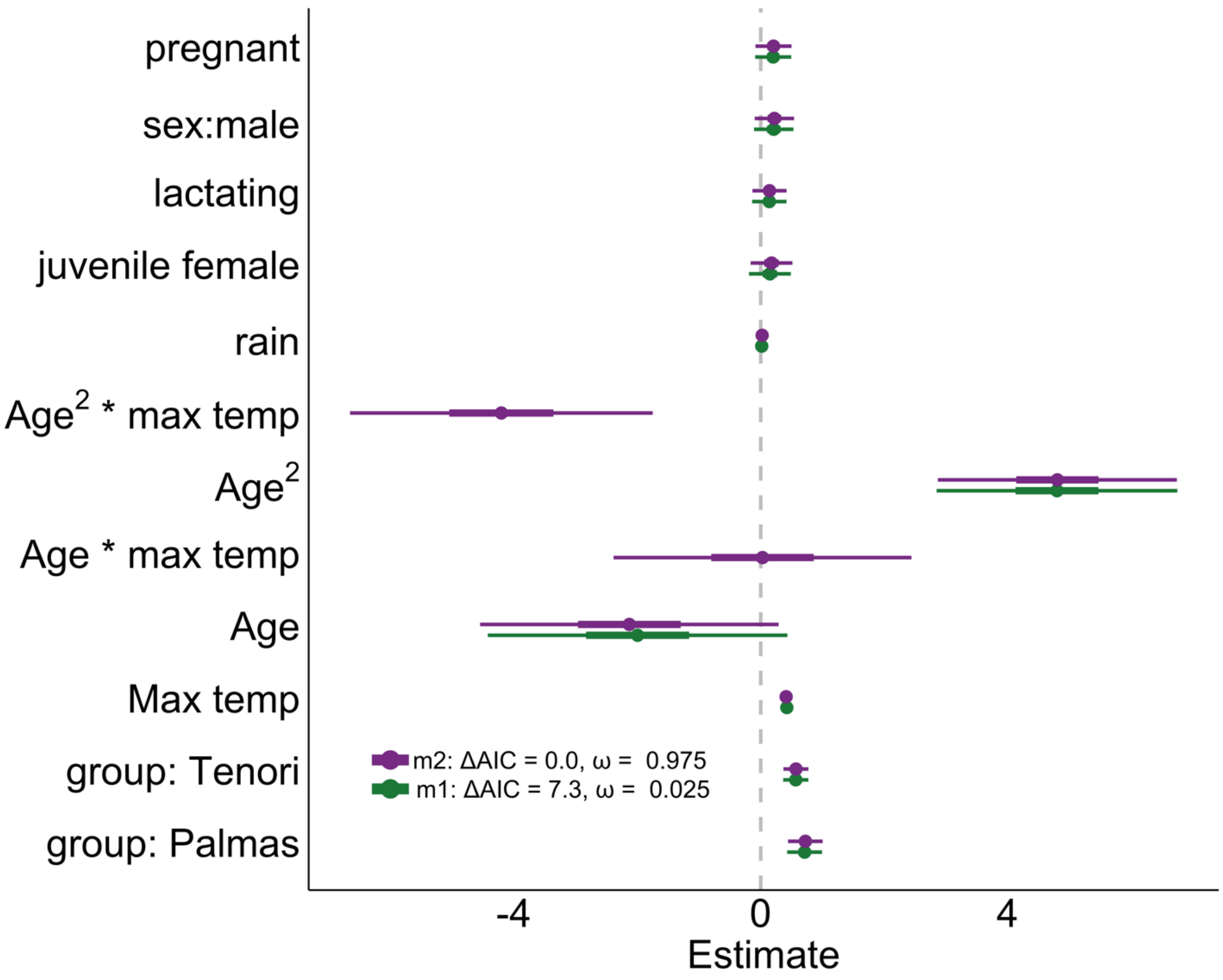

Fig S2. Comparison of generalized linear mixed models for models with greater than >0.001 % of the model weight. Includes all predictor variables used in each model. Similar results are seen across models and no reliable effect is seen for reproductive status, sex, or rain. Thick bars represent 50% CI, and thin bars represent 95% CI.

Table S1. Comparison of generalized additive model (GAM) results including and excluding the oldest individual in our population (34 years of age at the beginning of the study). The gam_filtered values exclude the oldest individual and are the results presented in the manuscript. The gam_full values include the oldest individual. The results do not change appreciably depending on the inclusion of the oldest individual.

model	variables	edf	ref.df	statistic	R-sq
gam_filtered	spline: age	3.67	4.15	7.13	0.364
	spline: max temp	4.79	5.84	17.41	
gam_full	spline: age	3.99	4.44	7.54	0.395
	spline: max temp	4.79	5.84	17.59	

Table S2. Comparison of generalized linear mixed model results including and excluding the oldest individual in our population. The models listed as ‘full’ include the oldest individual. The models listed as ‘filtered’ exclude the oldest individual and are the results presented in the manuscript. Here we compared both groups of models using AICc. For both groups of models, glmm_2 holds the majority of the model weight while glmm_1 holds the least of the model weight. The results do not change appreciably depending on the inclusion of the oldest individual.

model	dAICc	df	weight
glmm_2_full	0.00	11	60.67
glmm_1_full	0.87	9	39.33
glmm_2_filtered	0.00	15	97.50
glmm_1_filtered	7.33	13	2.50

Table S3. Comparison of generalized linear mixed model results for models with greater than >0.001 % of the model weight including and excluding the oldest individual in our population. The models listed as 'full' include in oldest individual. The models listed as 'filtered' exclude the oldest individual and are the results presented in the manuscript. The beta coefficients and std. errors do not change appreciably depending on the inclusion of the oldest individual.

model	predictor	estimate	std.error
glmm_2_full	Age squared	4.54	0.92
glmm_2_full	max temp	0.42	0.05
glmm_2_full	Rain	0.02	0.04
glmm_2_full	Age squared * max temp	-2.60	1.29
glmm_2_filered	Age squared	4.81	0.99
glmm_2_filered	max temp	0.41	0.05
glmm_2_filered	Rain	0.02	0.04
glmm_2_filered	Age squared * max temp	-4.20	1.25

Table S4. Comparison of the generalized additive model with temperature averages at different time intervals before sample collection to identify best fitting temperature predictor. Maximum and minimum temperature predictors show congruent results. For both temperature measurements, temperature averages 15 days before sample collection is the best fitting time period given the AICc and model weight.

min_t_model	AICc	weight	max_t_model	AICc	weight
days15	0.00	0.32	days15	0.00	0.35
days16	0.71	0.23	days16	0.91	0.22
days14	1.38	0.16	days14	1.47	0.17
days17	2.10	0.11	days17	2.50	0.10
days18	2.78	0.08	days18	3.26	0.07
days19	4.32	0.04	days19	4.85	0.03
days13	4.94	0.03	days13	5.15	0.03
days20	5.80	0.02	days20	6.29	0.02
days21	7.93	0.01	days21	8.33	0.01
days28	9.62	0.00	days28	9.84	0.00
days12	14.18	0.00	days12	13.80	0.00
days35	17.81	0.00	days35	18.00	0.00
days11	20.02	0.00	days11	19.32	0.00
days10	21.64	0.00	days10	20.98	0.00
days9	22.81	0.00	days9	22.04	0.00
days5	23.12	0.00	days5	22.56	0.00
days7	26.86	0.00	days7	25.50	0.00
days2	27.13	0.00	days2	26.74	0.00
days3	27.95	0.00	days3	27.31	0.00
days1	28.05	0.00	days1	28.17	0.00

Table S5. Summary of results from the fitted generalized linear mixed models results for models with greater than >0.001 % of model weight. Results include the beta coefficient (estimate) and standard error for each variable included in each model. AICc scores and model weights are also provided.

model	predictor	estimate	std.error	dAICc	df	weight
glmm_1	group: Palmas	0.71	0.14	7.33	13	2.5
	group: Tenori	0.57	0.10			
	Age	-1.99	1.24			
	Age squared	4.80	0.99			
	Rain	0.02	0.04			
	Juvenile female	0.15	0.17			
	Lactating	0.14	0.14			
	Sex:Male	0.21	0.16			
	Pregnant	0.20	0.15			
	max temp	0.42	0.05			
glmm_2	group: Palmas	0.72	0.14	0.00	15	97.5
	group: Tenori	0.57	0.10			
	Age	-2.13	1.23			
	Age squared	4.81	0.99			
	max temp	0.41	0.05			
	Rain	0.02	0.04			
	Juvenile female	0.17	0.17			
	Lactating	0.14	0.14			
	Sex:Male	0.22	0.16			
	Pregnant	0.21	0.15			
	Age * max temp	0.03	1.23			
	Age squared * max temp	-4.20	1.25			
